# Sustainable Blended Cements—Influences of Packing Density on Cement Paste Chemical Efficiency

**DOI:** 10.3390/ma11040625

**Published:** 2018-04-18

**Authors:** Yaniv Knop, Alva Peled

**Affiliations:** 1 Department of Civil Engineering, Ariel University, Ariel 40700, Israel; 2 Department of Structural Engineering, Ben-Gurion University of the Negev, Beer-Sheva 653, Israel; alvpeled@bgu.ac.il

**Keywords:** sustainability, packing density, surface area, blended cement, hydration, carbonation, limestone

## Abstract

This paper addresses the development of blended cements with reduced clinker amount by partial replacement of the clinker with more environmentally-friendly material (e.g., limestone powders). This development can lead to more sustainable cements with reduced greenhouse gas emission and energy consumption during their production. The reduced clicker content was based on improved particle packing density and surface area of the cement powder by using three different limestone particle diameters: smaller (7 µm, 3 µm) or larger (70 µm, 53 µm) than the clinker particles, or having a similar size (23 µm). The effects of the different limestone particle sizes on the chemical reactivity of the blended cement were studied by X-ray diffraction (XRD), thermogravimetry and differential thermogravimetry (TG/DTG), loss on ignition (LOI), isothermal calorimetry, and the water demand for reaching normal consistency. It was found that by blending the original cement with limestone, the hydration process and the reactivity of the limestone itself were increased by the increased surface area of the limestone particles. However, the carbonation reaction was decreased with the increased packing density of the blended cement with limestone, having various sizes.

## 1. Introduction

Concrete is the most widespread building material in the industrialized world, and cement is considered as one of the most important components in the concrete mixtures. However, cement highly affects greenhouse gas emissions, as the production of each ton of cement releases almost one ton of CO_2_ into the atmosphere [1,2]. Globally, the production of cement contributes to at least 5–7% of CO_2_ emissions [3]. Furthermore, cement production requires a kiln at high temperature of approximately 1500 °C (e.g., high energy consumption). Therefore, the development of more sustainable cements with less CO_2_ emission and energy consumption is required to meet the demand for sustainability that will allow minimal impact on the environment. One common direction is the development of blended cement by partial replacement of the cement with mineral additives. Besides the ecological and environmental benefits, blended cements can also have economic benefits due to the use of low-cost mineral additives which in many cases are also waste products. Furthermore, such blended cements can also exhibit improvements in performance if properly blended and designed. Two main types of mineral additives are commonly used: (i) reactive materials, such as fly ash [4], slag [5,6], and metakaolin [7]; and (ii) inert materials, such as limestone—one of the most attractive additives, because it is considered natural, available, and economical. According to EN197-1 [8], all 27 common types of cement may contain 5% minor additional components (MACs), most typically limestone. Moreover, there are four types of cement that allow for a higher percentage of limestone replacement in two different ranges: CEM II/A-L and CEM II/A-LL (6–20% limestone); CEM II/B-L and CEM II/B-LL (21–35% limestone).

From a cement performance point of view, the most significant effect of limestone powder is on the hydration reaction rate of the cement components. Fine limestone particles act as nucleation centers for the hydration reaction [6,9,10,11]. Therefore, by increasing the surface area of blended cement using fine-particle limestone, the number of nucleation regions grows, causing the hydration rate and degree to increase. It was shown by Kadri et al. [12] that finely-ground limestone fillers promote the heterogeneous nucleation of hydrates, significantly accelerating hydration. Tsivilis et al. [13] showed that after one day of curing, an increase in limestone additives causes a relative increase in non-evaporable water, indicating a higher degree of hydration. Knop et al. [14] showed that by the increased surface area of the limestone powder in the blended cement, the setting times of the blended cement were reduced. It was also shown by Kumar et al. [15] that an increase in the fineness of either the cement or the limestone—or a greater limestone content—generally act to increase the chemical reaction rate. 

Another effect limestone has on blended cement systems is the “filler effect”. The partial replacement of the original cement by fine additives having high surface areas may cause a negative effect on the blended cement by increasing the water demand for maintaining constant workability [16]. However, it was also shown that the combination of additives in a blended cement increases its packing density [17]. An effect on the packing density was also shown by Knop et al. [18]; blended cement with limestone additive and combined particle sizes attained a greater packing density than single-sized limestone-blended cement. In addition, this effect of the limestone size and content on the packing density was simulated by a mathematical model [19]. The same effect was also observed by Vuk et al. [9], who showed that the addition of fine limestone powder (2% residue on a 90-mm sieve) decreases the water demand necessary for standard consistency and also decreases the initial and final settings, as measured by the Vicat method.

Yet another effect is related to the reactivity of limestone with the clinker minerals or with the hydration products. Although no significant effect of the limestone on cement performance has yet been attributed to the reactivity of the limestone, there are several studies showing that limestone is not a completely inert additive. Ramachandran [20] tested the hydration of the tri-calcium silicate (C_3_S) and tri-calcium aluminate (C_3_A) phases individually. It was found that the addition of limestone increases the amount of calcium hydroxide because of the increased hydration rate. The more limestone added, the greater the effect. This is particularly significant at the beginning of the hydration process. Using differential thermogravimetry (DTG), it was also shown that some of the limestone is consumed during hydration [20]. Tri-calcium aluminate (C_3_A) reacts with calcium carbonate to form two types of calcium carboaluminate: the high carbonate form, 3CaO·Al_2_O_3_·3CaCO_3_·32H_2_O, and the low carbonate form, 3CaO·Al_2_O_3_·CaCO_3_·12H_2_O. When both gypsum and limestone are added to blended cement, ettringite formation is accelerated by the addition of calcium carbonate [21]. Lothenbach et al. [22] provide experimental observations that monocarbonate—instead of monosulfate—is stable in the presence of limestone. According to Kakali et al. [23], the hydration products in C_3_S and C_3_A pastes containing CaCO_3_ may be identified by powder diffraction. In addition, the hydration of C_3_S is accelerated, and the formation of some carbosilicate is observed [23].

As discussed above, diverse investigations on limestone-blended cements are reported in the literature; however, less information is available on the influences of the packing density of the blended cement particles, which involve the particle size distribution and particle surface areas. Such parameters can affect the chemical reactions of the cement paste, and thus the overall performance of the cementitious system. Those influences and the involved chemical mechanisms are less well-known.

The goal of this research was to develop sustainable blended cements by partial replacement of the clinker amount with limestone. Limestone was chosen for reasons besides its environmental benefits, as it is considered natural, available, and economical. The idea behind this research was to develop blended cements with controlled surface area and particles packing density by using limestone powder of three different particle diameters: smaller or larger than the clinker particles or having a similar size, to maximize the chemical efficiency of the blended cement.

## 2. Materials and Methods

CEM I 52.5 R was partially replaced by limestone powders (>99.8% CaCO_3_) with varying particle sizes. The chemical composition of the original cement is presented in Table 1.

Five different limestone powders, representing several particle diameters—smaller than, larger than, or similar-in-size to the original CEM I, with an average particle diameter of 17 µm—were examined. The particle size distribution (PSD) of all the tested powders (limestone and original cement) was shown by Knop et al. [14]. The mean particle size and surface area of the original cement and the five limestone powders, having a specific gravity of 2.7 gr cm^−3^, are given in Table 2.

Cement replacements were investigated using limestone–cement mixtures in which the limestone powders comprised 0%, 20%, and 35% of the mixtures (by mass), where 0% represents a reference system of the original cements without the addition of limestone. The limestone powders of various sizes and the original cement were mixed using a tubular mixer for 20 min. Several testing methods were used to study the properties of the powders and the fresh cement pastes.

### 2.1. Powders

Surface area and particle size distribution were examined for the five different limestone systems and for the original reference cements. The surface area of each individual powder was determined using the Brunauer–Emmett–Teller (BET) technique with N_2_. The surface areas of the original cement and limestone powders were calculated by multiplying the cumulative relative weight of each powder by the surface area of the individual component in the powder mixture. The particle size distribution was determined by laser diffraction scattering (CSI-100, Ankersmid, Nijverdal, The Netherlands).

### 2.2. Fresh Cement Pastes

At the fresh stage, two test methods were employed:

(1) The heat of hydration was measured by isothermal calorimetry—TAM Air by TA instruments (New Castle, DE, USA) to study the heat flow from the moment the blended cement was mixed with water. All the samples were mixed with a constant water-to-cement ratio (0.4).
(2) The workability of the fresh cement paste was determined based on normal consistency. Each cement powder—blended or original—was mixed with the amount of water needed to obtain a normal consistency according to EN-196-3 [24]. These pastes were then used to study the properties at the harden stage, as described in the following section.

### 2.3. Hardened Cement Pastes

Several test methods were used to test the hydration degree and rate and to evaluate the mineral compositions over time in the hardened stage. After each sample (i.e., original cement or original cement + limestone) reached normal consistency, it was placed in water at 20 ± 1 °C until the time of testing (up to 28 days, depending on the test method). At each testing time, fragments were immersed in acetone for 1 h to remove the water and then kept in an oven for 120 min. at 60 °C. Immediately after this procedure, the specimens were kept in a vacuum until testing.

The test methods employed were:

(1) The amount of the non-evaporable water in the hydrated pastes was determined in order to evaluate the degree of hydration (also at late ages, from 2 h to 28 days). For cement pastes, the degree of hydration may be determined as follows: the hydration of 1 g of anhydrous cement produces 0.23 g of non-evaporable water. The LOI (loss-on-ignition) non-evaporable water content is determined by the relative mass loss between 105 °C and 1000 °C, corrected for the LOI caused by decarbonation [25,26].
(2) Quantitative phase analysis of X-ray diffractions (XRDs), using the Rietveld method, was applied to determine the mineral content of the cement pastes, including both the original cements and the cements mixed with limestone at the age of 28 days after casting. XRD analysis was performed using XRD—EMPYREAN X-Ray Diffractometer (CuKα radiation, 45 kV, 40 mA, PANalytical, Almelo, The Netherlands) in a scanning range of 7° to 53° in 2θ at an internal of 0.020°.
(3) Thermogravimetry and differential thermogravimetry (TG/DTG) was used for the determination of the weight loss due to the dehydration of calcium hydroxide and the decarbonation of calcium carbonate in the hardened sample from 2 h to 28 days after casting, using a TGA—TA Instruments Q500 (New Castle, DE, USA). The samples were heated in the 20 °C to 1000 °C range at a constant rate of 10 °C/min in an atmosphere of N_2_.

## 3. Results and Discussion

### 3.1. The Effect of Particle Size on Hydration Reaction

**Figure 1:**
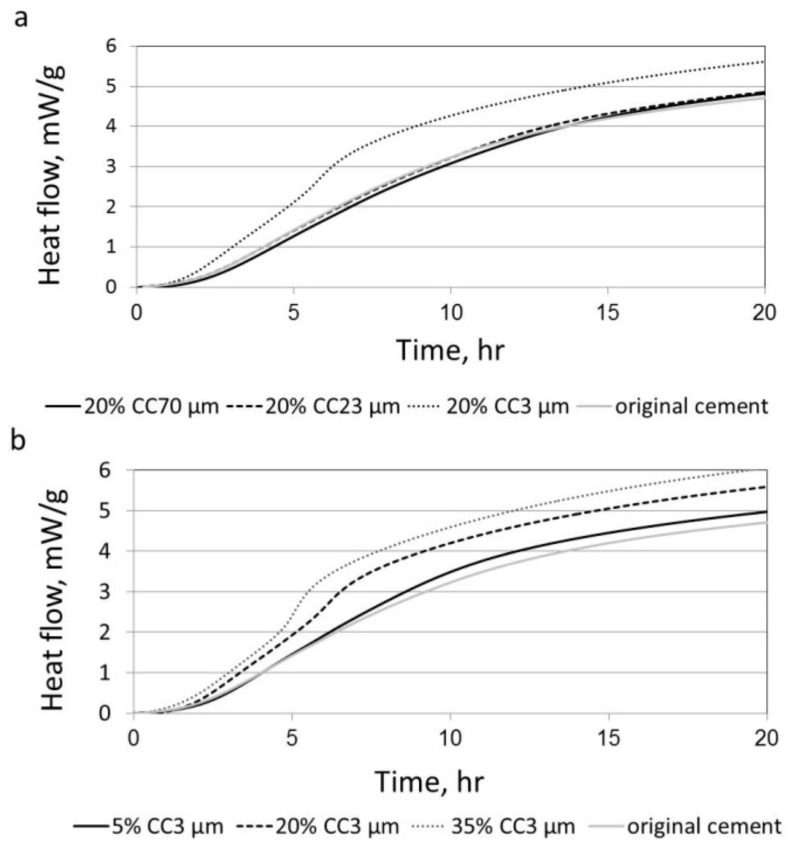
Evolution of the hydration heat for the original cement and blended cements with limestone having: (**a**) various sizes with 20% limestone content; and (**b**) fine powder (CC3 µm) with different limestone contents.

The influence of limestone particle sizes on the hydration processes at the initial stages in the different blended cements with various particle sizes and in the original cement was studied by isothermal calorimetry (Figure 1a). Heat flow was divided by the weights of the original cement contents in the blended cements. Note that the fine limestone-blended cement (CC3 µm) was the only blend showing significantly different behavior. A greater heat flow was obtained for the fine limestone-blended cement than in the other tested cements with medium and large particles and when compared to the original cement. The normalized hydration heat of the blended cements containing fine limestone powder (CC3 µm) in various quantities is presented in Figure 1b, along with that of the original cement. This figure indicates that hydration heat increased with increased limestone content, despite the higher percentage of limestone additive (dilution effect) in the blended cement. Moreover, at all three levels of replacement (5%, 20%, 35%), the heat released was greater than in the original cement. Therefore, according to Figure 1, partial replacement of the original cement with fine limestone powder increased the hydration rate at the initial stage, although this decreased the active component in the blended cement (original cement). A similar effect on the hydration reaction, mainly at the initial stages, was reported in the literature [6,9,10,11,18].

**Figure 2:**
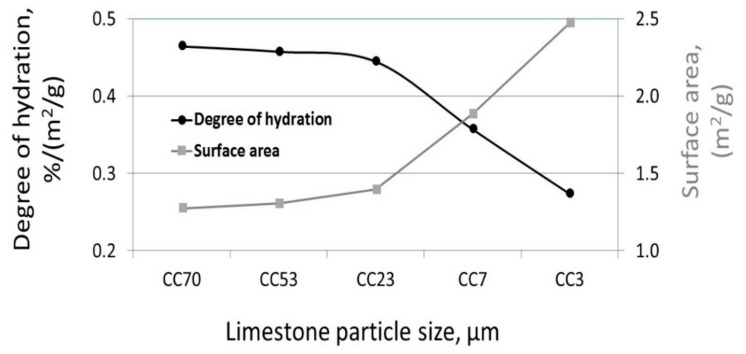
Normalized hydration degree to the surface area and the surface area of the combined-sized 20%-limestone blended cement, 1 day after casting.

It is shown in Figure 1 that the increase in surface area increased the degree of hydration. However, as was shown by Knop et al. [14], the packing density of blended cement with fine limestone powder (CC3 µm) decreases due to agglomeration; empty spaces are left between the agglomerated particles, as was clearly shown by SEM observations. As such, the surface area added by the limestone in the blended cement was not effective enough to act as nucleation centers in the blended-cement paste (clearly seen in Figure 2). Figure 2 shows the ratio between the hydration degree calculated by LOI (%), and the calculated surface area (m^2^/g) of the blended cement with 20% limestone in various sizes (CC70 µm, CC53 µm, CC23 µm, CC7 µm, CC3 µm) 1 day after casting. As seen, the normalized hydration degree to the surface area was insignificantly different for blended cements with large and medium particles (CC70 µm, CC53 µm, CC23 µm). However, a significant decrease in the normalized hydration degree was obtained for blended cements with small particles and higher surface areas (CC7 µm, CC3 µm), compared to the blended cements with large and medium particles (CC70 µm, CC53 µm, CC23 µm). The greatest decrease was found in the blended cement with the finest limestone powder (CC3 µm). Therefore, the “calculated surface area” cannot represent the “effective surface area” of the blended cement, because of the reduced surface area due to agglomeration of the fine particles in the cement paste.

**Figure 3:**
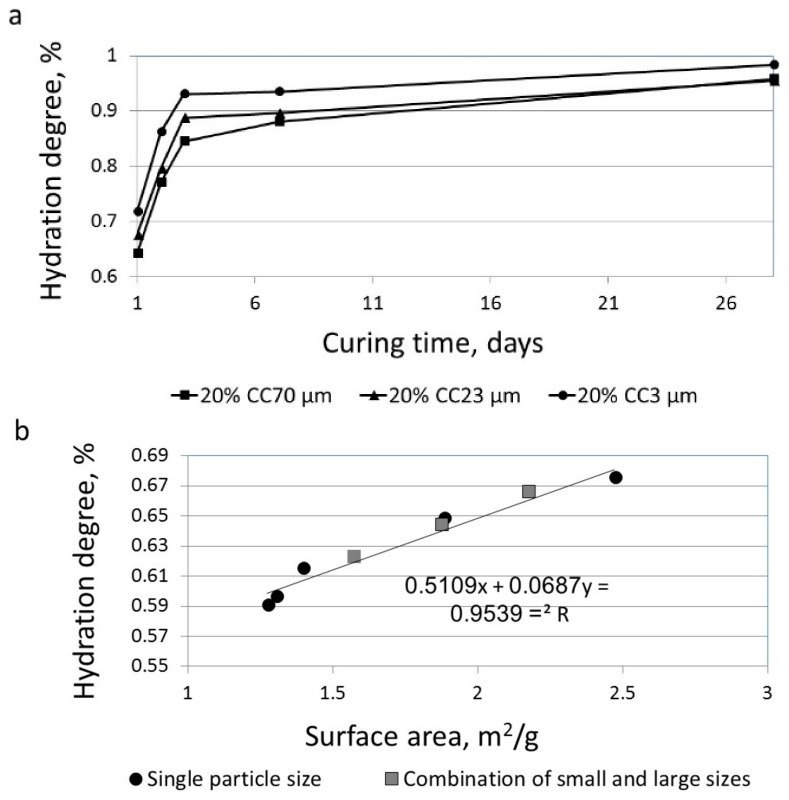

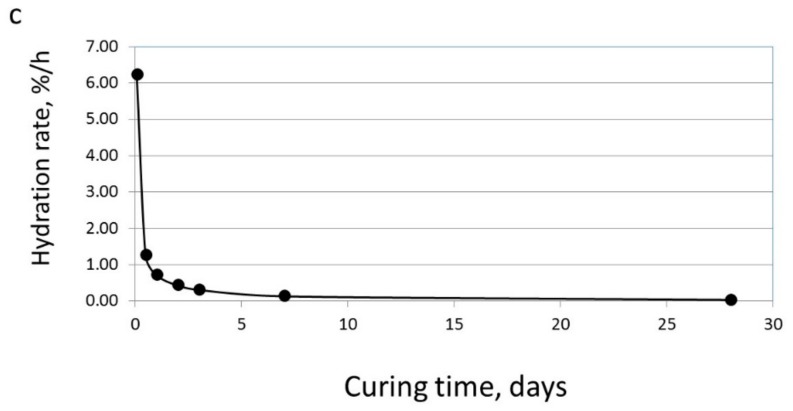
The effect of limestone particle size on the hydration reaction (20% replacement): (**a**) hydration degree from 1 to 28 days after casting; (**b**) linear regression of the hydration degree vs. particle surface areas at 24 h; (**c**) rate of hydration (%/h) vs. age of limestone-blended cement with 20% small particles (CC3 µm).

The degrees of hydration of several blended cements were calculated based on the amounts of non-evaporable water measured by the LOI technique at different ages. This was done to allow for the evaluation of the influences of the different limestone particle sizes at more advanced stages (late ages). Limestone-blended cements with three different particle sizes—CC70 µm, CC23 µm, and CC3 µm—were investigated at the ages of 1, 2, 3, 7, and 28 days after casting. Higher degrees of hydration were calculated for blended cements with fine limestone powder and high surface area (at all the tested ages between 1 and 28 days) than were found in the blended cements with larger particle sizes and lower surface areas (Figure 3a). No significant increase in the hydration degree was observed 7 days after casting in the case of limestone-blended cements having large (CC70 µm) and medium (CC23 µm) sized particles. In other words, the finer the particle size and the larger the surface area, the higher the hydration level also at late stages. 

This trend was even more pronounced when correlating the hydration values measured 24 h after casting with particle surface area by linear regression (R^2^ = 0.9539). The greater surface area of the blended cements resulted in higher degrees of hydration (Figure 3b). Here, the results of two systems are presented: limestone-blended cements with single-sized particles (either CC70 µm, CC53 µm, CC23 µm, CC7 µm, or CC3 µm) and limestone-blended cements having combined particle sizes, large and small (CC70 µm + CC3 µm) at various ratios. However, as shown in Figure 3c, the hydration rate (hydration degree %/h) was high during the initial stage of hydration, but became significantly reduced 12 h after casting, as was also shown by Ramachandran [19]. The trends observed in the current work correlate well with those published results. 

It may be concluded that the quantity of the hydration products increased at early ages—as well as at later ages—with the increased surface area of the limestone particles, due to the increased number of nucleation centers in the blended-cement paste. Yet, note that no significant increase in the hydration degree was observed for limestone-blended cements having large- and medium-sized particles after the age of 7 days. However, the effective surface area of the blended cement with the fine limestone powder was reduced due to the agglomeration of those fine particles.

### 3.2. The Effect of Particle Size on the Amount of Ca(OH)_2_

Cement paste is known as a porous material [27], enabling the penetration of various substances into its bulk after hardening. One of the gases that can diffuse into the hardened cement paste is CO_2_. By the presences of moisture, the CO_2_ can react with the Ca(OH)_2_—one of the main hydration products—by carbonation reaction. The corrosion of steel bars may start when the protective oxide layer is destroyed either by chloride penetration or because of the reduction of the pH value of the concrete below 9. The reduction of the alkalinity can be caused by the carbonation reaction [28]. Therefore, the corrosion protection of reinforcing steel by high alkalinity due to the presence of Ca(OH)_2_ is significantly reduced if the carbonation reaction entered up to the steel bar location, leading to reduction in the service life of the concrete component [29]. One way to reduce the carbonation reaction and its penetration depth into the cement paste is by using SCMs (supplementary cementing materials). If additives such as silica fume and fly ash are used, they react with the Ca(OH)_2_ by pozzolanic reaction, and accordingly the carbonation process and depth is reduced [28,29]. However, less is known about the influences—if valid—of inert materials such as limestone on the carbonation reaction. Hence, in this study the influences of limestone and its particle sizes on the amount of Ca(OH)_2_ were evaluated. Based on the calorimetric measurements presented in Figure 1, it is suggested that the hydration rate is increased in limestone-blended cement with small additive particles, which may imply a greater amount of Ca(OH)_2_ within the paste. However, this measurement provides general information on the hydration degree and rate, but does not provide specific information about each hydrate component content.

**Figure 4:**
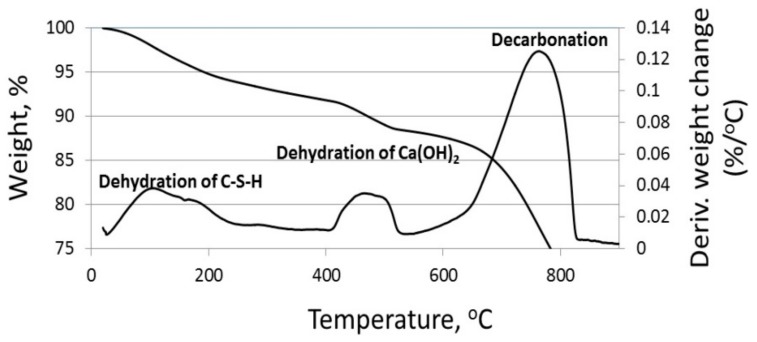
Thermogravimetric and differential thermogravimetric (TG/DTG) curves of a sample with 20% CC70 µm at an age of 28 days. The dehydration of Ca(OH)_2_ occurs in the temperature range of 400–500 °C.

TG/DTG measurements were thus performed to assess the specific amount of calcium hydroxide in the tested cements with the different particle sizes. Figure 4 shows representative TG and DTG curves of limestone-blended cement with large-sized particles (CC70 µm) at the age of 28 days. Here, three main regions were observed, representing three different reactions [22]:

- Up to 300 °C: removal of water from hydrated products.
- 400–500 °C: dehydration of calcium hydroxide.
- 600–800 °C: decarbonation of calcium carbonate.

The dehydration of the calcium hydroxide at 400–500 °C is according to the following chemical reaction (Equation (1)):

(1) $$\mathrm{Ca}{\left(\mathrm{OH}\right)}_{2}\;\to \mathrm{CaO}+{\;H}_{2}O.$$

**Figure 5:**
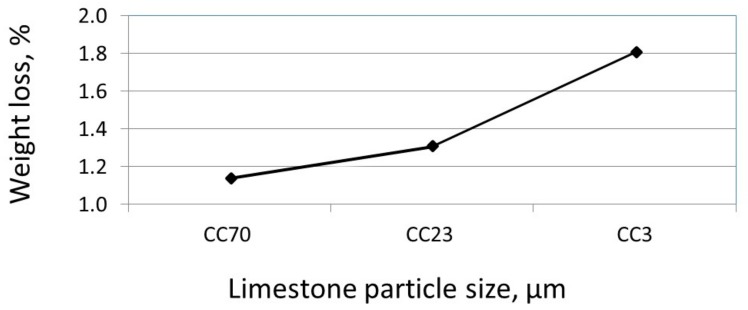
Weight loss due to the dehydration of Ca(OH)_2_ in limestone-blended cements with 20% CC70 µm, CC23 µm, and CC3 µm, 2 h after casting.

Figure 5 shows the weight loss due to Ca(OH)_2_ dehydration vs. the age of several blended cements with different limestone particle sizes at different ages. In general, at 2 h after casting, a higher weight loss (due to Ca(OH)_2_ dehydration) was observed in the fine limestone-blended cement with the higher surface area than in the large-sized limestone-blended cements, as expected. The greater Ca(OH)_2_ content was due to a greater hydration reaction promoted by the additional surface area in the blended cement, thanks to the presence of the fine limestone (see Figure 1a).

**Figure 6:**
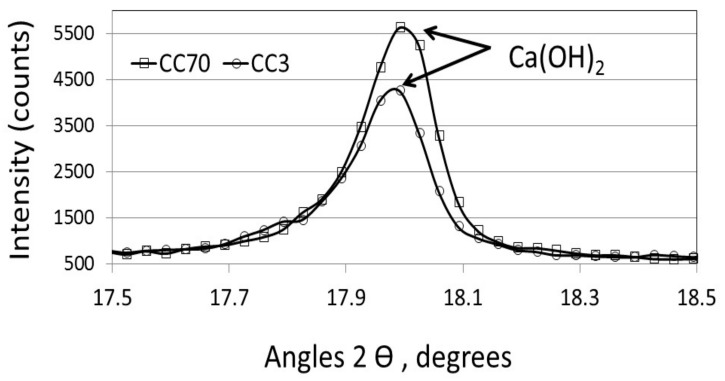
XRD patterns of limestone-blended cements with CC70-µm and CC3-µm sized particles 28 days after casting (magnified region of angular (2θ = 17.5–18.5)).

Despite the correlation between the surface area and the amount of calcium hydroxide found in Figure 5, an opposite trend was observed in XRD patterns after 28 days for the different blended cements with large and small limestone particle sizes—CC70 µm and CC3 µm (Figure 6). Higher peak intensity (i.e., greater Ca(OH)_2_ content), was clearly observed for the large-sized limestone-blended cement with a low surface area (CC70 µm), as compared to the fine limestone-blended cement with a higher surface area (CC3 µm).

Based on the above results, it may be said that the influence of limestone particle size on the amount of Ca(OH)_2_ was inconsistent, showing different trends at early age (2 h, Figure 5) and late age (28 days, Figure 6) for the same blended cement. These inconsistent trends in the amount of Ca(OH)_2_ suggest that an additional chemical reaction may also be involved (besides the hydration reaction of the cement components with water). One possible influence on the amount of Ca(OH)_2_ in the cement paste may be due to its reaction with the CO_2_ diffused into the paste from the environment; i.e., a carbonation reaction may take place in the cement, as shown in Equation (2) [29]:

(2) $$\mathrm{Ca}{\left(\mathrm{OH}\right)}_{2}+{\;\mathrm{CO}}_{2}\to {\mathrm{CaCO}}_{3}+{\;H}_{2}O.$$

The carbonation reaction rate (Equation (2)) may be affected by two main factors: the reactivity of the calcium hydroxide with CO_2_, and the diffusion rate of the CO_2_ into the hardened paste. The diffusion of CO_2_ may be affected by the paste density (higher density causes lower CO_2_ diffusion). According to Talukdar et al. [29], the intrinsic diffusion rate at which CO_2_ is transported into the cement paste depends on the size and the connectivity of the pore system. As the water/cement ratio is lowered, the pore system becomes finer and less connected, leading to lower transport rates and less effective diffusivity. Therefore, it is essential to determine the effect of both the powder surface area (which promotes the hydration reaction; Figure 1), and of the packing density of the cement paste (which has a significant effect on the diffusivity of CO_2_ within the cement paste).

**Figure 7:**
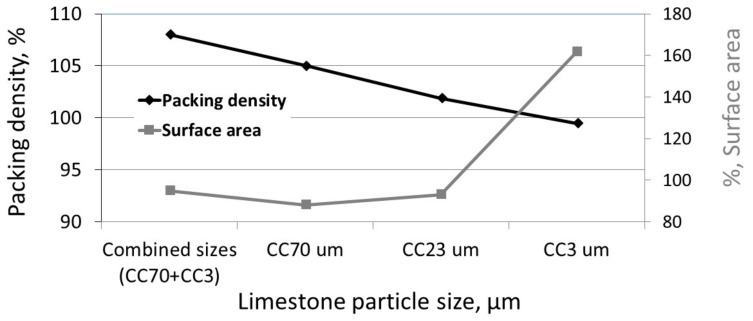
Surface area (%) and packing density (%) of limestone-blended cement with single-sized and combined-sized additive particles (the lines are only for visual guides).

To evaluate the effects of surface area and packing density on the amount of Ca(OH)_2_, the surface areas and the packing densities of limestone-blended cements with single-sized and combined-sized particles were determined. The initial density (packing density) of the fresh-blended cement paste was calculated by measuring the water demand necessary for reaching a normal consistency, according to EN196-3 [14]. Figure 7 presents the surface areas and the initial packing densities of limestone-blended cements with single-sized particles (CC70 µm, CC23 µm, CC3 µm) and with combined-sized particles (17% CC70 µm + 3% CC3 µm) relative to the surface area and packing density of the original cement. Note that with increased particle size, the surface area of the single-sized limestone-blended cement decreased, while the packing density increased. It was also observed that the packing density of limestone-blended cement with combined-sized particles was significantly greater than that of a single-sized blend, although its surface area was similar to that of limestone-blended cement with medium-sized particles (CC23 µm). Additionally, at later ages, large-sized limestone-blended cement obtained greater density, as reported earlier in [14].

**Figure 8:**
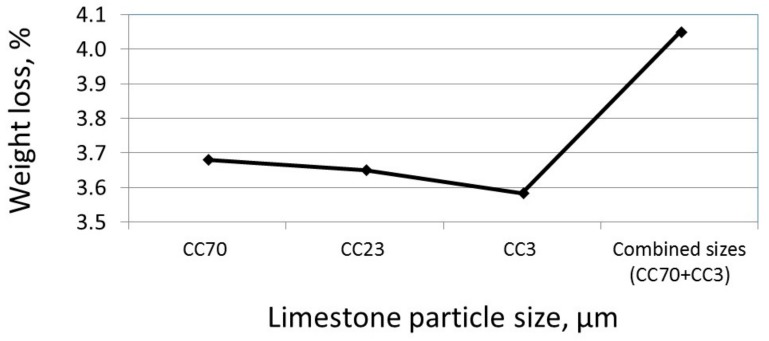
Weight loss due to the dehydration of Ca(OH)_2_ in limestone-blended cements with 20% CC70 µm, CC23 µm, CC3 µm, and combined-sized particles (17% CC70 µm + 3% CC3 µm) 28 days after casting.

To evaluate the amount of calcium hydroxide at the age of 28 days and to understand the effect of the packing density on the amount of calcium hydroxide, the dehydroxylation of Ca(OH)_2_ was measured for single-sized (CC70 µm, CC23 µm, CC3 µm) limestone-blended cements and for combined-sized particles (17% CC70 µm + 3% CC3 µm). The weight losses due to the dehydroxylations of the calcium hydroxide in both the single-sized and combined-sized blended cements at the age of 28 days are presented in Figure 8. At 28 days, a higher amount of Ca(OH)_2_ was observed in single-sized blended cements with lower surface areas and greater packing densities, although it was expected that with the increased surface area, the amount of Ca(OH)_2_ would increase due to the increased level of hydration. This trend of increased Ca(OH)_2_ in larger particle-sized blended cements correlates with the XRD results (Figure 6), also measured at 28 days. One may also see in Figure 8 that combined-sized blended cements sustained the greatest weight loss due to Ca(OH)_2_ dehydration, despite having a surface area similar to that of blended cement with medium-sized particles (CC23 µm, Figure 7). 

According to Talukdar et al. [27], and based on the results shown in Figure 7 and Figure 8, it is suggested that the increased packing density of the blended cement reduced the transport rates and lowered the effective diffusivity of the CO_2_ into the paste. At 28 days, the paste density was significantly higher than at an age of 2 h, suggesting a lower CO_2_ diffusion rate. Thus, at 28 days, the CO_2_ diffusion is expected to be a significant factor—one which determines the reaction rate of the calcium hydroxide with the CO_2_. Therefore, in blended cements having high particle sizes and high packing densities (Figure 7), higher amounts of calcium hydroxide were measured (Figure 8). The same trend (of high calcium hydroxide) was obtained for combined-sized limestone-blended cements, compared to single-sized ones. In cases of combined-sized blended cements, the packing densities were significantly higher than those of single-sized ones, leading to a significant amount of calcium hydroxide at 28 days (Figure 8).

### 3.3. The Effect of Particle Size on the Reactivity of Limestone Powder

Limestone is considered as an inert component in the blended cement, but there is substantial evidence that it is not a completely inert component. The hydration process yields additional products, mostly created by the reaction of the limestone with the C_3_A phase [20]. However, the size of the limestone particles can further influence such reactivity, which was less explored. In this study, the reactivity of the limestone, depending on its particle size, was investigated by means of two test procedures: XRD, to evaluate the monosulfate content; and TG/DTG, to measure the calcium carbonate content.

**Figure 9:**
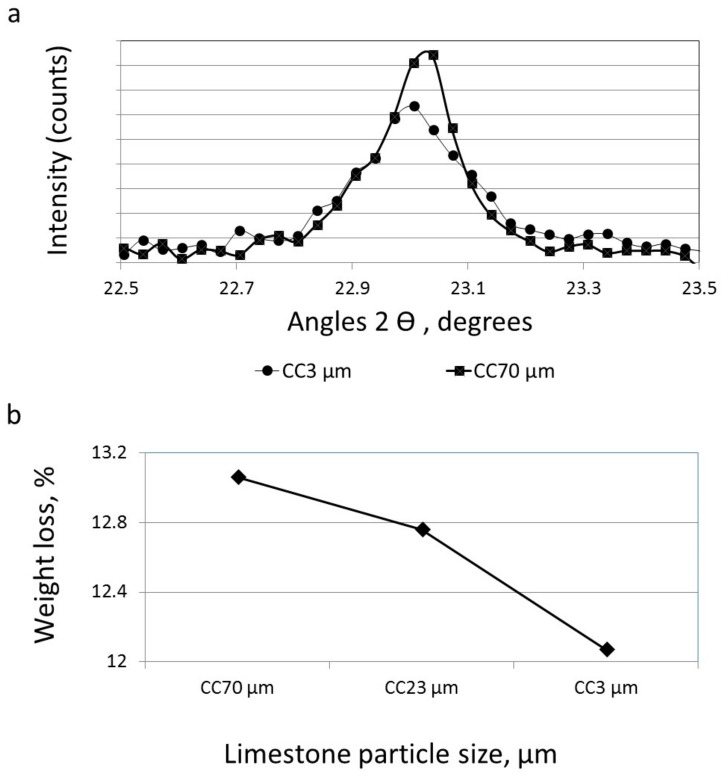
(**a**) XRD patterns of limestone-blended cement with CC70-µm and CC3-µm sized particles 28 days after casting (magnified region of angular (2θ = 22.5–23.5) related to monosulfate); (**b**) weight loss using TG/DTG due to the decarbonation of CaCO_3_ in limestone-blended cements with particle sizes CC70 µm, CC23 µm, and CC3 µm, 3 days after casting.

The zone related to the monosulfate (taken by XRD, following Kakali et al. [23]) is magnified in Figure 9a. Despite the small intensity differences, it can be seen that the smallest limestone particle (CC3 µm) blended cement (with the highest surface area) was observed to have the lowest peak intensity, while higher peak intensities were seen for the large-particle (CC70 µm) blended cements. Thus, the particle size of the limestone powder influenced the monosulfate content within the hardened paste. Less monosulfate is formed within pastes made of fine-particle limestone (i.e., having high surface areas). Lothenbach et al. [21] suggested that the formation of monocarbonate—instead of monosulfate—is more stable in the presence of limestone. According to Kakali et al. [23], calcium aluminate monocarbonate is preferably formed instead of monosulfate in the presence of CaCO_3_. 

The decarbonation of CaCO_3_ following Equation (3) (the amount of calcium carbonate) was measured by TG/DTG for three blended cements having different limestone particle sizes—CC70 µm, CC23 µm, and CC3 µm—at the age of 3 days (Figure 9b). The degree of decarbonation diminished along with a decrease in limestone particle size and increased surface area; i.e., a low limestone content in a fine-particle blended-cement paste.

(3) $${\mathrm{CaCO}}_{3}\;\to \mathrm{CaO}+{\;\mathrm{CO}}_{2}$$

Based on the different amounts of CaCO_3_ in the blended cements (Figure 9b), it may be concluded that limestone particles did react during the hydration process in cement pastes, while this reaction was dependent on the surface area of the limestone (i.e., particles size). The chemical reaction mechanism is still not well understood; however, it is evident that fine limestone particles (high surface area) exhibited greater reactions than large-sized limestone particles (low surface area).

**Figure 10:**
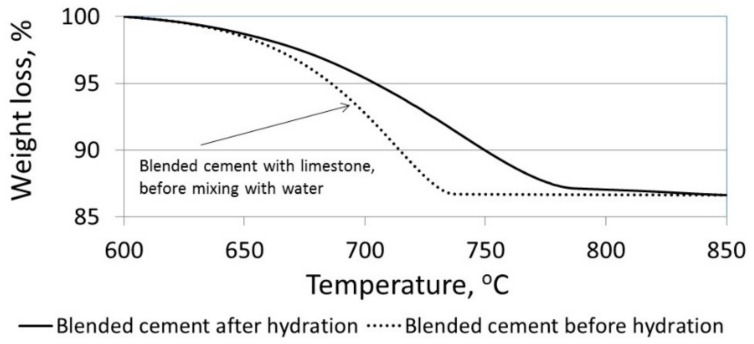
Weight loss of limestone-blended cement with fine particles (CC3 µm) in the “virginal” stage, before mixing with water and after 3 days of hydration.

As shown in Figure 8, the amount of calcium hydroxide was reduced by the decrease in limestone particle size, suggesting that the calcium hydroxide may have reacted with the CO_2_ by means of a carbonation reaction. However, the reactivity of the limestone was greater as the particle size decreased (as was shown in Figure 9), and thus a lower limestone content was measured. The weight loss (by TG) in limestone-blended cements with small particles (CC3 µm) in the “virginal” stage (i.e., before mixing the powder with water), and after 3 days of hydration in the 600–850 °C temperature range, is presented in Figure 10. Note that a similar weight loss was obtained for dry-blended cement before hydration and after 3 days of hydration, meaning that the amount of limestone was the same before hydration (in the “virginal” stage) and after 3 days of hydration. These facts do not fit with the previous findings, indicating that the limestone particles were reacting during the hydration process (Figure 9). Based on the results obtained, it may be stated that the total amount of calcium carbonate within the hardened paste is affected by two parallel, but opposite, reactions. On one hand, it is affected by the increased reaction of the calcium hydroxide with the CO_2_; i.e., by the carbonation rate as more calcium carbonate is added to the hardened paste. On the other hand, it is influenced by the increased reactivity of the limestone particles as more calcium carbonate is consumed. Therefore, the quantity of calcium carbonate (limestone) after 3 days of hydration remains the same as the pre-hydration amount (Figure 10).

## 4. Conclusions

In this paper, blended cements with reduced clinker amount were examined. The reduced clicker content was based on improved particles packing density and surface area of the blended cement powder by using various particle diameters of limestone. 

It was found that by blending the original cement with limestone, the hydration process, carbonation, and reactivity of the limestone were affected depending on limestone particles’ surface area and the related powder packing density. 

The hydration rate of the clinker components including the amount of calcium hydroxide was increased with the increase in blended cement surface area due to the increased number of nucleation centers in the blended-cement paste at early age. However, at 28 days, the amount of calcium hydroxide was reduced as the packing density decreased, due to carbonation reaction, as CO_2_ diffusion into the hardened paste was rather easy. It can be concluded that the amount of calcium hydroxide was affected by two parallel but opposite reactions. On one hand, it was affected by the increased reaction of the calcium hydroxide with the CO_2_; i.e., by the carbonation rate as more calcium carbonate was added to the hardened paste. On the other hand, it was influenced by the increased hydration rate, caused by the increased surface area of the limestone powders.

Although they are considered as inert materials, the limestone particles were found to react in the blended-cement paste. This reaction was found to be influenced by the limestone particle size. Higher amounts of limestone reacted in the small-sized limestone-blended cements. As such, it may be concluded that limestone is not a completely inert component in blended cements, and that its reactivity is dependent on particle size and the corresponding surface area.

It can be concluded that besides the environmental and economic benefits of the blended cements developed in the current work, due to the replacement of the non-environmental and high-cost clinker with a friendlier environment and low-cost limestone material, such inert additive—if properly designed—can affect the chemical reaction and efficiency of the blended cement pastes. Greater chemical reaction is expected to improve the overall behavior of the cementitious paste and the related mortar and concrete products in terms of mechanical performance as well as their durability, which can further benefit their sustainability.

## Figures and Tables

**Table 1:** Chemical composition of the original cement.

Component	CaO	SiO_2_	Al_2_O_3_	Fe_2_O_3_	MgO	TiO_2_	K_2_O	Na_2_O	P_2_O_5_	Mn_2_O_3_	SO_3_
%	65.07	18.96	4.5	2.46	1.16	0.36	0.33	0.21	0.32	0.30	2.86

**Table 2:** Mean particle size and surface area (Brunauer–Emmett–Teller, BET) of the tested powders.

Property	Unit	Cement CEM I	CC70 µm	CC53 µm	CC23 µm	CC7 µm	CC3 µm
Mean particle size	µm	17.02	70.28	53.40	23.01	7.07	2.99
Surface area (BET)	m^2^/gr	1.53	0.23	0.39	0.85	3.29	6.22
